# sexual offenders : Epidemiological and Criminological Profile

**DOI:** 10.1192/j.eurpsy.2022.2087

**Published:** 2022-09-01

**Authors:** O. Charaa, W. Bouali, L. Brahmi, A. Haj Mohamed, R. Ben Soussia, L. Zarrouk

**Affiliations:** Hospital Tahar sfar Mahdia, Department Of Psychiatry Mahdia, Mahdia, Tunisia

**Keywords:** Sexual offender, sexual agression

## Abstract

**Introduction:**

Sexual assault is a major problem in Tunisian society. There is no definitive typology of the characteristics of those who sexually assault. A great diversity of sexual aggression behaviors and different motivations can be described.

**Objectives:**

It is about a retrospective survey, achieved from data of Medical Expertise of the sexual offenders achieved in psychiatric departement of hospital of mahdia. This study revealed 18 cases during the period from January 2010 to December 2020.

**Methods:**

The objective of the work was to describe the epidemiological and criminological profile of the sexual assaults.

**Results:**

Mean age of the sample was 40 years [30-61]. Aggressors were almost exclusively males, have medium socioeconomic status and without a regular job. Fifty percent of the perpetrators had a psychiatric diagnosis: bipolar disorder (27.7%), schizophrenia (11.1%), antisocial personality disorder (5.5%) and intellectual disability (5.5%). Indecent assault (27.7%) was the most frequent aggression then the rape (22,2%). Thirty three per cent of the victims were minor.Among these expertised patients, 72% were considered responsible for their actions and only one was considered irresponsible.

**Conclusions:**

Studies on the characteristics of sexual offenders have concluded to the profile of the young, single and unemployed male, but it can’t be a commun profile.

**Disclosure:**

No significant relationships.

